# Generation and Transfer of Entanglement in a Circular Spin System

**DOI:** 10.3390/e28040393

**Published:** 2026-04-01

**Authors:** Vinh Le Duc, Joanna K. Kalaga, Wiesław Leoński

**Affiliations:** 1 Tinh Gia 3 High School, Nghi Son District, Thanh Hóa 42700, Vietnam; 2 Quantum Optics and Engineering Division, Institute of Physics, University of Zielona Góra, Prof. Z. Szafrana 4a, 65-516 Zielona Góra, Poland

**Keywords:** spin system, entanglement, mixed states, negativity, linear entropy, Ising model

## Abstract

We consider an Ising-type model of six interacting spins in a closed circular configuration. We discuss two scenarios in which the system is initially in either an entangled or a product state. In the first scenario, we analyze how entanglement is transferred among pairs of spins and how the coupling strength affects such a transfer. In the second scenario, we demonstrate that the creation of a strongly entangled state depends on the coupling parameters. We demonstrate that careful selection of the coupling strength can increase the degree of entanglement generated in the system, as measured by negativity, and control which spin pair becomes strongly entangled. Additionally, the relationship between linear entropy—a measure of mixedness—and negativity, a measure of entanglement, is discussed.

## 1. Introduction

Quantum entanglement plays an important role in quantum processes such as quantum cryptography [1], quantum computing [2], quantum metrology [3], quantum communication [4], quantum information [5], and quantum machine learning [6]. The generation of quantum entanglement has been intensively studied in various systems, including cavity magno-mechanical systems [7,8,9], mechanical resonators [10,11,12,13,14], optical waveguides [15,16,17,18], Kerr nonlinear couplers [19,20,21,22,23,24], optical [25,26,27] and fermionic [28] lattices.

Entanglement also plays a crucial role in various problems in quantum information processing. In particular, information transfer could be observed in the so-called quantum Internet (qI). In the not-so-distant future, qI will offer capabilities that surpass the boundaries of traditional communication methods. In classical communication systems, light transmission through optical fibers can lead to photon loss, which can degrade signal quality. To overcome this problem, direct amplification of optical signals is often used. However, when we are dealing with qI, such amplification is not possible to realize, as the non-cloning theorem shows that such a procedure is not possible to perform. This poses a challenge for long-distance transmission. One solution is to deploy intermediate nodes, known as quantum repeaters [29], between distant nodes. Such repeaters can transmit quantum entanglement by continuously generating strong entanglement at the nodes and then transmitting it as soon as the nodes hold the entangled qubits.

Various systems have been proposed in the literature for transferring quantum entanglement. For example, such transfer can be found in the circuit QED models, which involve microwave cavities [30], optomechanical systems [31,32,33,34,35], one-dimensional Bloch electron [36], and PT-symmetric-like systems [37]. Additionally, the transfer of quantum entangled states has also been intensively studied in Heisenberg models of XXX1/2-spin chains placed in an external homogeneous magnetic field [38,39,40,41,42]. In certain systems, quantum entanglement can be transferred with high accuracy. Some studies have reported that maximum transfer efficiency can be as high as 0.998 [41] and 0.99 in [42]. The transfer of quantum entanglement through a 1/2-spin XXZ chain forming a quantum wire whose ends are connected to qubits by switchable coupling was also investigated by Banchi et al. [43]. The study demonstrated that the transfer of quantum entanglement through the quantum wire remains high-quality even as the number of spins and the length of the wire increase. The transfer of quantum entanglement is also faster in this type of spin chain compared to the XXX spin chain. However, it should be noted that such a transfer is not perfect, particularly as the number of spins involved in this model increases. On the other hand, an advanced model of two XXZ spin chains has also been considered by Ji et al. [44]. It appeared that, with increasing spin-chain length, quantum entanglement occurs randomly and remains small. Nevertheless, the duration of the presence of quantum entanglement was too short to be used as a resource for quantum information processing. Furthermore, the achieved entanglement was weak for a small number of spins.

In this paper, we study the generation and transfer of quantum entanglement in a circular spin Ising-like system. We assume that the system under consideration is isolated from any external bath and is finite in size. For such a system, we study the creation and transfer of quantum entanglement without accounting for complications arising from quantum criticality. Our results show that the produced entanglement between two spins can be almost maximal. We then examine the transfer of entanglement between spin pairs and demonstrate its high efficiency. This suggests that negativity can reach significant levels for various spin pairs, which is often accompanied by the simultaneous disappearance of entanglement in an initially entangled pair. For the purposes of this paper, we arbitrarily define a spin pair as strongly entangled if its negativity exceeds 0.9. In addition, we discuss the relations between the negativities describing the entanglement appearing in our model and corresponding to the linear entropies measuring mixedness of the states generated in the system.

In contrast to earlier studies devoted to entanglement dynamics in spin systems, such as the analysis of homogeneous Ising chains in [45], investigations of noncollinear interactions in finite rings reported in [46], or focused on state transfer in spin chains with inhomogeneous external magnetic fields in [41], this paper concentrates on a finite, closed, Ising-like ring with a deliberately introduced local imbalance in the spin–spin couplings introduced to control entanglement redistribution. The generation and transfer of entanglement in spin chains has been extensively studied, including the effects of anisotropy and magnetic fields in parallel Heisenberg chains [47], junction geometries [48], dissipative environments [49], or quantum behavior in extended models with two- and four-spin interactions [50]. In contrast, little attention has been given to systematic, physically transparent mechanisms that enable the selective enhancement of strong pairwise entanglement in strictly finite and isolated ring geometries. Our results demonstrate that controlled asymmetry in nearest-neighbor couplings provides an efficient and versatile tool for redistributing entanglement across the ring. This allows negativity to approach near-maximal values for selected spin pairs while simultaneously suppressing correlations elsewhere. By explicitly quantifying the interplay between negativity and linear entropy for dynamically generated mixed states, we further clarify how entanglement enhancement correlates with state mixedness in a closed, finite system. Thus, the present study offers a focused and technically well-defined contribution that complements, rather than overlaps with, existing literature, and refines the understanding of controllable entanglement engineering in finite quantum spin networks.

Moreover, it should be emphasized that the model presented here, due to its geometry, could be applied in studies related to quantum information processing; for instance, to those devoted to quantum repeaters [51] where transfer of the entanglement plays a crucial role. Additionally, the model’s specific geometry allows it to be treated as a hexagonal system that can also serve as a brick subsystem within more complex systems exhibiting graphene-like geometry. It provides perspectives on further investigation of entanglement transfer in such systems.

The paper is organized as follows. First, we introduce the model in Section 2. We then study the transfer of entanglement for different values of the spin–spin coupling (Section 3). In Section 4, we concentrate on the generation of entanglement for two cases: when the symmetric initial state is assumed, and that corresponding to an asymmetric one. Furthermore, in Section 5, we analyze the relationships between the linear entropy and the negativity, describing the entanglement that appears in our model. Finally, in Section 6, our findings and conclusions are presented.

## 2. The Model

Here, we analyze the transverse spin-Ising system [52]. Our model consists of six spin-1/2 particles labeled 1 to 6. Each spin interacts with its nearest neighbors along the x-axis defined by the line that connects them. The first and last spins also interact with each other. Consequently, all particles form a ring chain. The interactions between spins are described by the spin–spin coupling parameter $J_{i,i+1}$. In addition, all spins are exposed to a uniform external magnetic field characterized by the strength parameter *h*. For our analysis, we assume that the magnetic field is applied in the *z*-direction. Such a system is described by the following Hamiltonian:

(1) $$H=\sum_{i}^{N}J_{i,i+1}S_{i}^{x}S_{i+1}^{x}+h\sum_{i}^{N}S_{i}^{z},$$

where $S_{i}^{x}=\frac{1}{2}\sigma_{x}$ and $S_{i}^{z}=\frac{1}{2}\sigma_{z}$ are the local spin operators defined by the Pauli operators $\sigma_{x}$ and $\sigma_{z}$, respectively. The first term of the Hamiltonian (1) represents the interactions between spins, whereas the second one describes the coupling with a transverse magnetic field.

For the sake of simplicity, we assume that the system is isolated and does not interact with the external environment. Therefore, we can use a wave-function approach to analyze the system’s time evolution. Throughout this work, we set ℏ=1. To find the state of the system |ψ(t)〉 at the time *t*, we apply the unitary evolution operator $U=e^{-iHt}$ to the initial state |ψ(t=0)〉:

(2) |ψ(t)〉=U|ψ(t=0)〉.

## 3. The Transfer of Entanglement

**Figure 1:**
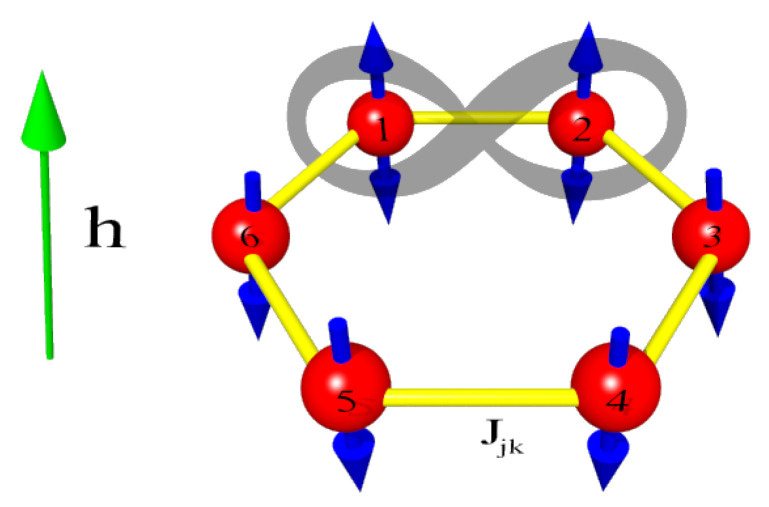
Model of the spins forming the hexagon representing a circular spin system. The red spheres with blue arrows represent the spins, and the yellow segments symbolize the spin–spin couplings *J*. The vertical green arrow shows the direction of the external magnetic field of the strength *h*. The spins 1 and 2 are initially in the Bell-like maximally entangled state.

In this section, we examine how the entanglement is transferred from one pair of spins to the others. Therefore, we will assume that two spins, labeled 1 and 2, are initially maximally entangled $\frac{1}{\sqrt{2}}\left({|↑↓\rangle }_{12}-{|↓↑\rangle }_{12}\right)$, whereas the other spins are pointed down (see Figure 1). In such a case, the wave-function describing the system for the time t=0 can be expressed as:

(3) $$|\psi \left(t=0\right)\rangle =\frac{1}{\sqrt{2}}\left({|↑↓\rangle }_{12}-{|↓↑\rangle }_{12}\right)⊗{|↓\rangle }_{3}⊗{|↓\rangle }_{4}⊗{|↓\rangle }_{5}⊗{|↓\rangle }_{6}.$$

To quantify the entanglement between two spins labeled *i* and *j*, we use negativity, an entanglement measure derived from the Peres–Horodecki positive partial transpose (PPT) criterion [53,54]. According to this criterion, a bipartite quantum state is entangled if the partial transpose of its density matrix possesses at least one negative eigenvalue. The negativity is then defined as

(4) $$N\left(\rho_{ij}\right)=\max\left(0,\;-2\min_{l}\lambda_{l}\right)$$

where $\lambda_{l}$ are the eigenvalues of the partially transposed reduced density matrix $\rho_{ij}$ [55]. Here, $\rho_{ij}$ is the reduced density matrix describing the two-spin subsystem, obtained from the full density matrix of the system by tracing out the other subsystems (the four remaining spins). The negativity $N(\rho_{ij})$ ranges from zero for separable states to one for maximally entangled states. As we discuss here, for two-qubit (two-spin) entanglement, the negativity is always a true measure of entanglement. Further, using the notation ij (or i−j), we denote that we describe the entanglement between spins *i* and *j*.

**Figure 2:**
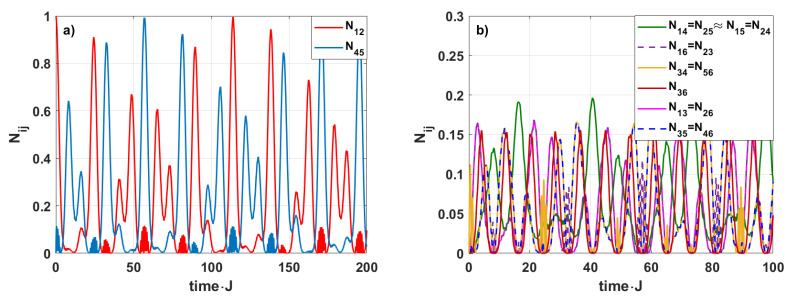
Time-evolution of the bipartite negativities $N_{ij}$ for h=1, J=0.25. Subfigure (**a**) shows the case in which strong entanglement is generated. Subfigure (**b**) corresponds to weak entanglement, for which the negativity does not exceed 0.2.

Assuming that all interaction parameters $J_{ij}$ are equal to each other $J_{ij}=J$, for the system initially prepared in the state (3), the entanglement is transferred between the spins in such a way that the initial maximal entanglement between spins 1 and 2 disappears and, at the same time, reappears between the spins 4–5 (see Figure 2a). In addition, over a longer period, the entanglement between the pair 1–2 recovers from its initial decay and approaches 1. This is a result of the system’s cyclical structure and unitary evolution. Concerning the other spin pairs, the entanglement does not exceed 0.2 (see Figure 2b). Changes in the interaction strength *J* do not affect the situation. The strongest entanglement is obtained for the pairs 1–2 and 4–5, whereas the entanglement corresponding to other pairs of spins is significantly weaker. Therefore, in the next step, we will vary the strength of the spin–spin interaction for only one spin pair, while keeping the other unchanged. We will study how this modification affects entanglement generation in the system.

**Figure 3:**
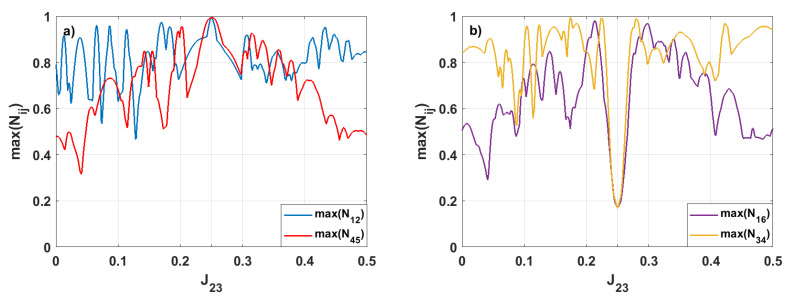
The maximal values of the bipartite negativities $\max(N_{ij})$ for various values of $J_{23}$ when the other coupling parameters are $J_{ij}=0.25$. We present only the results for which the negativities reach values $\max(N_{ij})\geq 0.9$ for the pairs of spins: (**a**) 1–2, 4–5, and (**b**) the pairs 1–6, 3–4.

Figure 3 shows the maximal values of the negativities $\max(N_{ij})$ that can be reached for various values of the interaction’s strength $J_{23}$—the strengths of other interactions remain unchanged and equal to 0.25.

As in the first case, strong entanglement is predominantly localized in the initially entangled pair 1–2, due to the ring geometry, partially mirrored in the pair 4–5 (see Figure 3a). Varying $J_{23}$ introduces a controlled local asymmetry that strongly affects the propagation of quantum correlations. Since spin 2 is directly connected to both spins 1 and 3, modifying $J_{23}$ alters the balance between the two competing pathways along which entanglement can spread around the hexagon. When $J_{23}$ is appropriately tuned, one propagation direction is favored. As a consequence, the entanglement initially localized in the pair 1–2 is preferentially redirected in a selected direction around the ring. This results in enhanced entanglement for pairs 1–6 and 3–4. In contrast, in the fully symmetric case with identical couplings, these pairs remain weakly entangled due to the uniform, nondirectional redistribution of quantum correlations.

**Figure 4:**
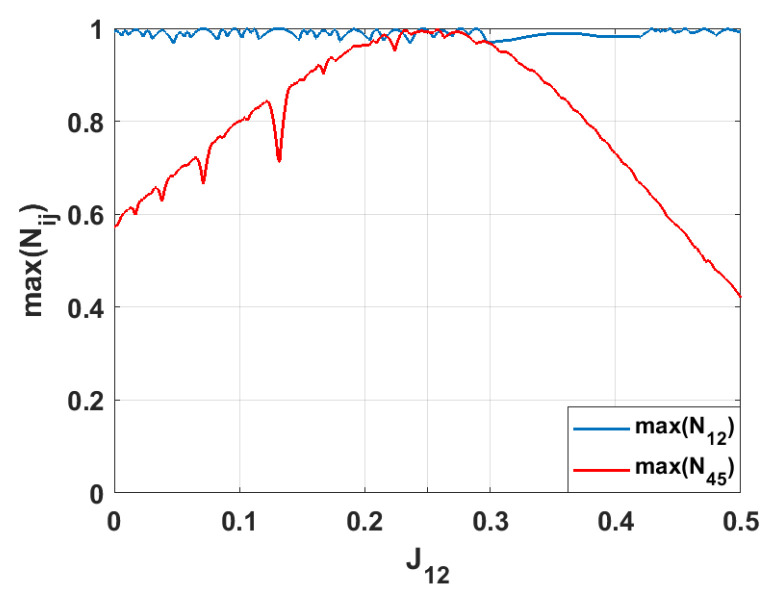
The maximal values of the bipartite negativities $\max(N_{ij})$ for various values of $J_{12}$. The remaining coupling parameters are $J_{ij}=0.25$. We present only the results for which the negativities reach values $\max(N_{ij})\geq 0.9$.

In contrast, varying the coupling strength $J_{12}$ does not lead to strong entanglement emerging in any spin pair besides the initially entangled pair, 1–2 (see Figure 4). Since spins 1 and 2 are in a maximally entangled state, changing $J_{12}$ does not create a new pathway for entanglement propagation. Instead, it increases the mismatch between this pair and the rest of the system. Consequently, the redistribution of quantum correlations is suppressed, and the entanglement in the symmetry-related pair 4–5 decreases as the difference between $J_{12}$ and the other couplings, $J_{ij}$, increases.

**Figure 5:**
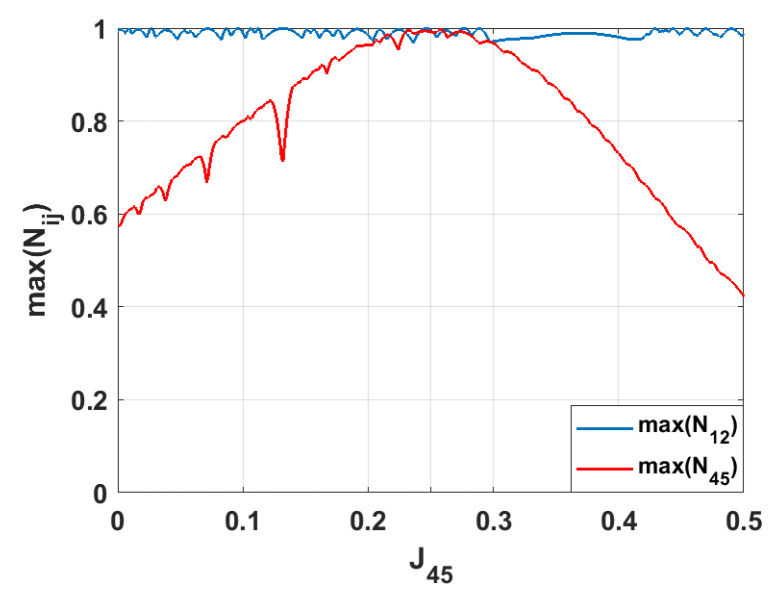
The same as in Figure 4, but for this case, $J_{45}$ is varied, whereas the other coupling parameters are J=0.25. We present only those negativities that reach values $\max N_{ij}\geq 0.9$.

Similar behavior is observed when varying the coupling $J_{45}$ (see Figure 5). Due to the rotational symmetry of the hexagonal geometry, the pair 4–5 is equivalent to the initially entangled pair 1–2. Consequently, tuning $J_{45}$ affects the system qualitatively the same way as tuning $J_{12}$, leading to no efficient transfer of strong entanglement to other spin pairs.

Thus, we see that strong entanglement can be transferred from the initially entangled pair 1–2 to different target spin pairs within the hexagonal system by appropriately manipulating the coupling strengths. The set of spin pairs exhibiting strong entanglement depends on which interaction is tuned. For example, varying $J_{34}$ generates strong entanglement ($\max N_{ij}\geq 0.9$) for pairs 1–2, 2–3, 4–5, and 5–6, whereas tuning $J_{56}$ favors pairs 1–2, 1–6, 3–4, and 4–5. Similarly, manipulating $J_{16}$ enhances entanglement for pairs 1–2, 2–3, 4–5, and 5–6.

These results demonstrate that strong entanglement can be transferred to various spin pairs by tuning a single coupling. However, this procedure must be performed carefully. An inappropriate choice of coupling strength may suppress the transfer instead, highlighting the need for careful parameter selection.

## 4. The Generation of Entanglement

In this section, we focus on the problem of whether our system can generate two-spin entangled states, assuming that the initial system’s state is a product state. For the first case, spins 1 and 2 are in the upward position for time t=0, while the others are pointed downward. The wave function for such a case can be written as:

(5) $$|\psi \left(t=0\right)\rangle ={|↑\rangle }_{1}⊗{|↑\rangle }_{2}⊗{|↓\rangle }_{3}⊗\ldots ⊗{|↓\rangle }_{6}=|↑↑↓↓↓↓\rangle .$$

In the following discussion, this state will be referred to as the *symmetric* one.

The second initial state under analysis, which is called *asymmetric* state, is of the form:

(6) $$|\psi \left(t=0\right)\rangle ={|↑\rangle }_{1}⊗{|↓\rangle }_{2}⊗{|↓\rangle }_{3}⊗\ldots ⊗{|↓\rangle }_{6}=|↑↓↓↓↓↓\rangle .$$

**Figure 6:**
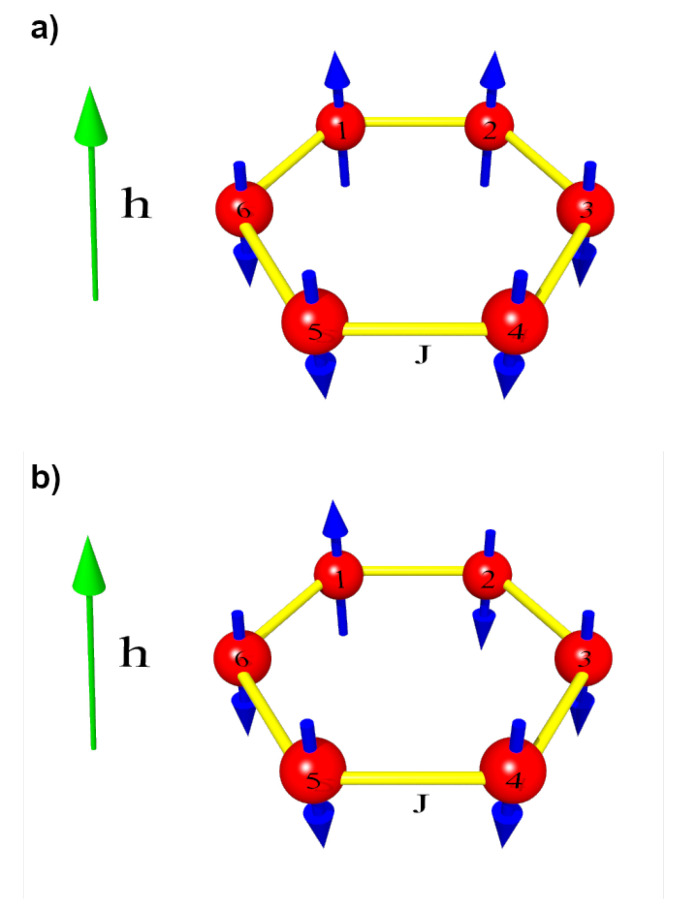
Initial orientation of the spins for (**a**) the symmetric and (**b**) asymmetric cases. The strength of interaction between two neighboring spins is denoted by *J*, whereas the external magnetic field strength is denoted by *h*.

When the system is in the state described by the above wave function, only the first spin is up, whereas the others are down. Both situations are depicted in Figure 6.

**Figure 7:**
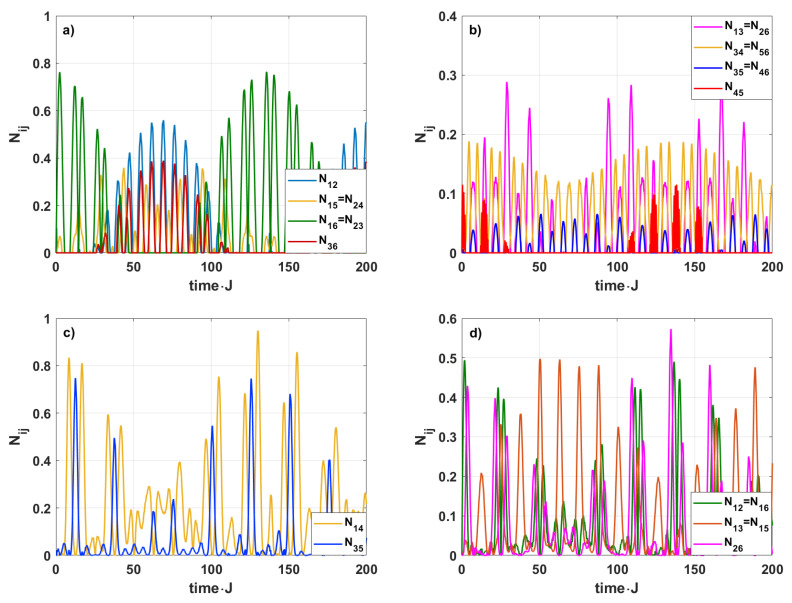
The time-evolution of the bipartite negativities $N_{ij}$ for h=1, J=0.25. The initial state is assumed to be symmetric (**a**,**b**) or, (**c**,**d**) asymmetric.

The terms “symmetric” and “asymmetric” refer to the symmetry properties of the entanglement dynamics, not just the structure of the initial product states—see Figure 7. In the initial configuration with two upward spins at t=0, the system is described as symmetric because the generated entanglement spreads symmetrically along the spin ring when the coupling strengths are uniform. Consequently, the negativities of the symmetry-related spin pairs evolve identically. For example, $N_{16}=N_{23}$ and $N_{15}=N_{24}$, and analogous relations hold for other equivalent pairs. In contrast, for the second initial configuration, such symmetry relations are only partially preserved (e.g., $N_{12}=N_{16}$ and $N_{13}=N_{15}$). To facilitate the subsequent discussion, we introduce the distinction between symmetric and asymmetric configurations, as there is a qualitative difference in the evolution of entanglement.

In Figure 7, we show the time evolution of the negativities $N_{ij}$ for both symmetric and asymmetric initial states when all couplings are $J_{ij}=0.25$. In the first case, when the initial state of the system is symmetric, some of the negativities reach the same value due to both symmetries—that of the system and that corresponding to the initial state. We can also see in Figure 7a,b that the entanglements 1–4 and 2–5 are not produced. Moreover, the entanglements 1–3, 2–6, 3–4, 5–6, 3–5, 4–6, and 4–5 are very weak, and the corresponding negativities do not exceed 0.2. In the symmetric case, only the strong entanglements for the spins 1–6 and 2–3 are generated. During the time interval, when the entanglements within the pairs 1–6 and 2–3 disappear after a few deaths and rebirths, we observe that the entanglement corresponding to the pair 1–2 becomes dominant. Unfortunately, this entanglement is not particularly strong—$\max N_{12}<0.6$. The terms “entanglement death” and “entanglement rebirth” denote finite time intervals during which the negativity vanishes. In the analyzed case, these intervals correspond to extended time windows. Moreover, the duration of the zero-entanglement intervals is not fixed and varies during the system’s evolution.

In the second case, when the initial state of the system is asymmetric, we can easily see in Figure 7c,d that only the entanglements 1−k (k=2,…,6), 2–6 and 3–5 appear. The entanglements for other spin pairs are not observed. At the beginning of the system’s evolution, due to the equal coupling between the spins, the entanglements 1–2 and 1–6 appear simultaneously with the same strength. Then, the other entanglements appear one after the other. Among these appearing entanglements, the entanglement between spins 1–4 is the strongest, and the maximum value of $N_{14}$ takes the value close to 0.95. Interestingly, there are no sudden-death or rebirth effects for the entanglement-generation processes appearing there. Moreover, we can see that $N_{35}$ reaches its maximum when $N_{14}$ is minimal; close to zero. That means that the entanglement 3–5 is maximally strong when the entanglement 1–4 disappears, and vice versa. The entanglement 1–4 reaches its maximum when the entanglement 3–5 practically disappears. For instance, at the first maximum of the negativity $N_{14}$, where $N_{14}=0.834$, the corresponding value of $N_{35}$ is only 0.023. Similarly, at the second maximum of *N*_14_, where $N_{14}=0.810$, the entanglement between subsystems 3–5 is further reduced, to $N_{35}=0.013$. These values suggest that the 3–5 entanglement is strongly suppressed whenever the 1–4 entanglement is maximal.

**Figure 8:**
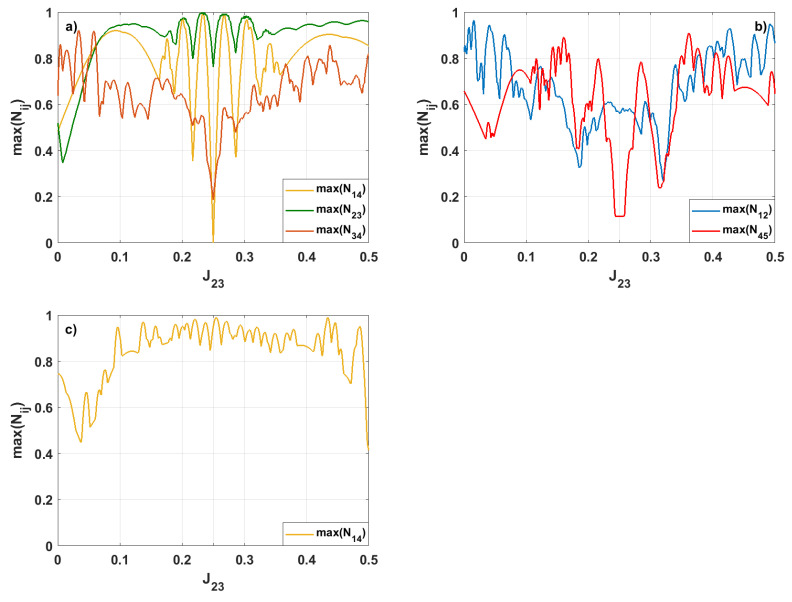
The maximal values of the bipartite negativities $N_{ij}$ for various values of $J_{23}$; the other coupling parameters are J=0.25. The initial state is (**a**,**b**) symmetric or (**c**) asymmetric. We present only those negativities for which $\max(N_{ij})\geq 0.9$.

In the next step, as in the previous section, we will study the influence of the coupling strength on the maximal value of the produced entanglement. Firstly, we concentrate on the dependence of the maximal values of the negativities on changes in the value of $J_{23}$, which characterizes the coupling between spins 2 and 3. The couplings between the other pairs of spins are equal to each other and are assumed to be $J_{ij}=0.25$. The studies are carried out for the two cases. For the first one, we assume the system’s initial state is symmetric, whereas in the second case, it is asymmetric. In both cases, we have concentrated only on the negativities $N_{ij}$ whose maximal values exceed values $\max N_{ij}\geq 0.9$. The results are shown in Figure 8.

When the system’s initial state is symmetric, we can see (Figure 8a,b) that only the negativities for the spin pairs 1–2, 1–4, 2–3, 3–4, and 4–5 reach values higher than 0.9. In addition, the maximal values of the negativities $\max(N_{ij})$ fluctuate strongly as the value of $J_{23}$ changes. As the value of $J_{23}$ increases, $\max(N_{12})$ fluctuates between 0.271 (for $J_{23}=0.320$) and 0.964 (for $J_{23}=0.016$). It should be pointed out that $\max(N_{23})$ first decreases from 0.520 to its minimal value, which is 0.347 (for $J_{23}=0.016$), and then increases to the value of 0.948 (for $J_{23}=0.110$). If we further increase the value of $J_{23}$ to 0.5, $\max(N_{23})$ varies between 0.764 and 0.990 for $J_{23}=0.25$ and $J_{23}=0.239$, respectively. Importantly, we can achieve an almost maximally entangled state for spins 2 and 3 even when the couplings in our system are unequal. In addition, we can also achieve strong entanglement between the spins 2 and 3 when $J_{23}=0.271$ and 0.303. For such the cases $\max(N_{23})$ reaches values of 0.994 and 0.984, respectively. For $N_{34}$ and $N_{45}$, we can easily see that their maximal values will be smallest when all the couplings in our system are equal to each other ($J_{ij}=0.25$). As the value of $J_{23}$ increases from 0 to 0.5, the maximal values of $N_{34}$ and $N_{45}$ also fluctuate. When $J_{23}=0.058$, $\max(N_{34})$ changes between 0.188 and 0.918, whereas $\max N_{(}45)$ between 0.115 and 0.909 for $J_{23}=0.362$. For the pair of spins 1 and 4, we can see that there is no entanglement between the spins 1 and 4 when all coupling parameters are equal. For the value of $J_{23}\neq 0.25$, the maximal value of $N_{14}$ fluctuates from zero to 0.996 (for $J_{23}=0.233$). Apart from this value of $J_{23}$, the almost maximal entanglement between spins 1 and 4 is also generated for $J_{23}=0.268$ ($\max(N_{14})=0.988$). It should be emphasized that the generation of entanglement between pairs of spins depends on the coupling parameter $J_{23}$. Its variation effectively breaks the homogeneity of the interaction network and controls the flow of quantum correlations along the ring. In particular, the dependence of $\max(N_{23})$ on $J_{23}$ demonstrates that an imbalance in the couplings can significantly increase the entanglement of spin pairs. Interestingly, almost maximal entanglement between spins 2 and 3 is achieved even for strongly non-uniform couplings, indicating that perfect symmetry is not a necessary condition for optimal entanglement generation. This behavior can be understood as a consequence of symmetry breaking. This process suppresses competition between different paths of entanglement propagation, localizing quantum correlations to specific spin pairs. A similar mechanism explains the strong entanglement between spins 1 and 4, which are not nearest neighbors. For uniform couplings, the entanglement between these spins is not present. However, deviations from $J_{23}=0.25$ allow for the attainment of a nearly maximal negativity. In general, these results show that controlled coupling imbalance provides an effective tool for engineering and selectively enhancing entanglement in interacting spin networks.

For the asymmetric initial state (Figure 8c), only $\max N_{14}$ reaches values higher than 0.9. In this case, the value of $\max(N_{14})$ fluctuates between 0.415 (for $J_{23}=0.5$) and 0.973 (for $J_{23}=0.271$). We can also see that, for the range of values of $J_{23}$ from 0.091 to 0.458, the value of $\max N_{14}$ is always greater than 0.8. Maintaining strong entanglement between spins 1 and 4, which is observed over a broad range of $J_{23}$ values, may result from the propagation of an initially localized excitation around the ring, as well as the constructive interference of amplitudes on opposite sides. Consequently, entanglement between spins 1 and 4 is favored, while entanglement between other spin pairs is hindered by competition and monogamy constraints.

**Figure 9:**
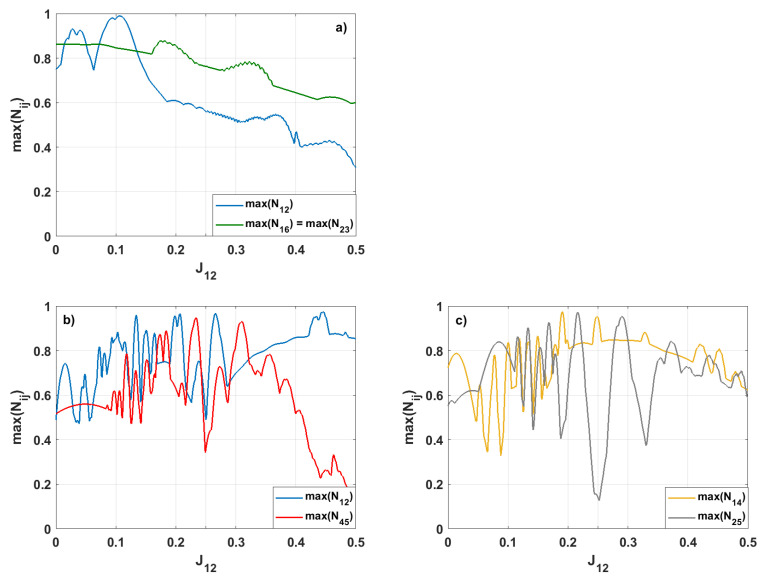
The maximal values of the bipartite negativities $N_{ij}$ for various values of $J_{12}$, the coupling parameters corresponding to other pairs of spins $J_{ij}=0.25$. The initial state is assumed to be (**a**) symmetric or (**b**,**c**) asymmetric. We present only those negativities that exceed the threshold $\max N_{ij}\geq 0.9$.

In the next step, we investigate how changing the $J_{12}$ value affects the generation of entanglement when the other couplings are J=0.25 (see Figure 9). We are interested in spin pairs for which $\max N_{ij}\geq 0.9$. Thus, when the system’s evolution starts from the symmetric state (Figure 9a), only the negativities $N_{12}$, $N_{16}$, and $N_{23}$ fulfill this condition. Due to the symmetry of the initial state of the system, the negativities $N_{23}$ and $N_{16}$ take the same value for each value of $J_{12}$. As we increase the value of $J_{12}$ from 0 to 0.5, $\max(N_{23})$ and $\max(N_{16})$ varies between 0.596 (for $J_{12}=0.494$) and 0.877 (for $J_{12}=0.174$). Moreover, these entanglements dominate for stronger interactions between the spins 1 and 2. When the coupling parameter $J_{12}$ is small, the entanglement between spins 1–2 is practically dominant. For $J_{12}=0.108$, we can obtain the almost maximal entanglement between these spins ($\max N_{12}=0.991$).

Figure 9b,c present the time-evolution of the negativities when the asymmetric initial state is assumed. In such a case, strong entanglement is produced for four pairs of spins. In consequence, for pairs 1–2, 1–4, 2–5, and 4–5 the negativity reaches values higher than 0.9. We can also see that adjusting the interaction strength between spins 1 and 2 determines which pair of spins exhibits strong entanglement. So for the interaction strengths $J_{12}=\left\{0.446,0.191,0.216,0.234\right\}$ the maximal values of negativity are $\max N_{12}=0.975$, $\max N_{14}=0.974$, $\max N_{25}=0.972$, $\max N_{45}=0.948$, respectively. Additionally, for the strong value of the interaction parameter $J_{12}$, the entanglement between the spins 1 and 2 is dominant, while the entanglement corresponding to other pairs of spins becomes weaker as the interaction strength increases.

**Figure 10:**
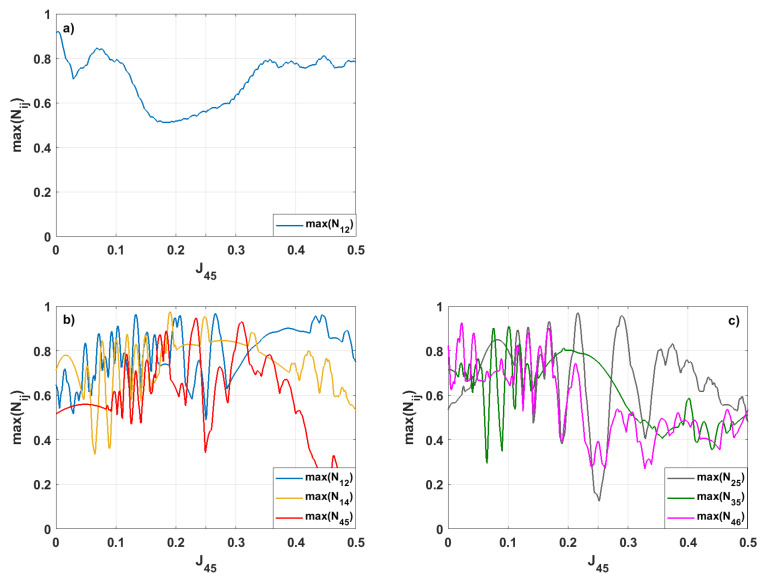
The same as in Figure 9 but now, for various values of $J_{45}$, the other coupling parameters are $J_{ij}=0.25$, and the initial state is (**a**) symmetric or (**b**,**c**) asymmetric. We present only the negativities exceeding values $\max N_{ij}\geq 0.9$.

Finally, we varied the value of $J_{45}$, keeping the other coupling parameters constant and equal to 0.25. The results for the initial symmetric and asymmetric states are presented in Figure 10. When the initial state of our system is symmetric, only $N_{12}$ reaches values greater than 0.9.

For the case where the system’s evolution starts from the asymmetric state (see Figure 10b,c) when $J_{45}=0.25$, except for the entanglement corresponding to the pair 1–4, the remaining entanglements are weak. That means that for the case of equal couplings between spins, there is no advantage for entanglement generation in our system. Importantly, by changing the strength of the interaction between spins 4–5, we can decide for which pair of spins we can obtain a strong entanglement. For instance, for $J_{45}=0.266$ we have $\max N_{12}=0.968$, for $J_{45}=0.190$ $\max N_{14}=0.976$, for $J_{45}=0.216$ $\max N_{25}=0.971$, for $J_{45}=0.101$ $\max N_{35}=0.909$, for $J_{45}=0.234$ $\max N_{45}=0.948$, and for $J_{45}=0.023$ $\max N_{46}=0.925$, respectively.

Our analysis shows that the entanglement dynamics in a hexagonal spin system depend on the combined effects of its geometry, the symmetry of its initial state, and coupling inhomogeneity. Breaking the symmetry by tuning the coupling parameters creates dominant entanglement among certain spin pairs. This enables the selective localization of quantum correlations on specific spin pairs. These results demonstrate that symmetry breaking via coupling imbalance is a practical and flexible tool for engineering and controlling entanglement in interacting spin networks. This may be relevant for spin-based quantum information and communication architectures.

## 5. Linear Entropy and Entanglement

The two-qubit quantum states generated in our system are mixed ones in general. Thus, the relationships between negativities and mixednesses are natural to analyze. To quantify the degree of mixedness, we use linear entropy defined with the application of the purity parameter [56]

(7) $$E\left(\rho \right)\equiv \frac{D}{D-1}\left[1-Tr\left(\rho^{2}\right)\right]\;,$$

where *D* is the dimension of the density matrix ρ. As we analyze the mixednesses of two-qubit states, the dimension D=4, and the linear entropy can be written as:

(8) $$E_{ij}=E\left(\rho_{ij}\right)\equiv \frac{4}{3}\left[1-Tr\left(\rho_{ij}^{2}\right)\right]\;,$$

where $\rho_{ij}$ is the reduced-density matrix describing the state of the given two-qubit subsystem.

We analyze the relationship between negativity and linear entropy to examine how mixedness limits the amount of entanglement that can be achieved in a system. It is well known that maximum entanglement can only be achieved with pure states, and increasing mixedness generally limits entanglement. However, we also investigate whether there are any unexpected features or limitations in the analyzed system.

**Figure 11:**
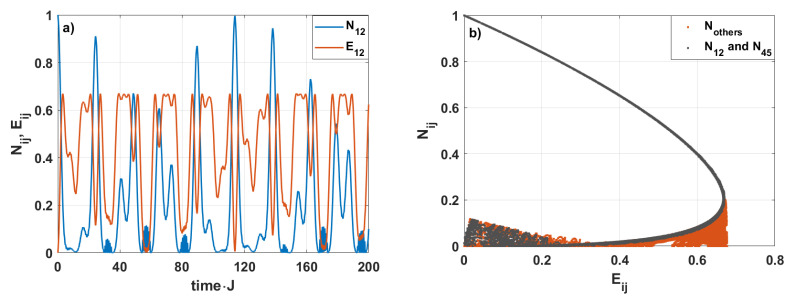
(**a**) The time evolution of the bipartite linear entropy and negativity for the spin pair 1–2, (**b**) the map of $N_{ij}$ versus $E_{ij}$ for all spin pairs and h=1 and $J_{ij}=0.25$.

Figure 11a depicts the time evolution of the negativity and linear entropy parameters for the spin pair 1–2 for the case already presented in Figure 2. As expected, the generation of strongly entangled states is strongly related to the decreasing values of the linear entropy. Comparing the time-evolution of $N_{12}$ and $E_{12}$, we see that the maximal values of negativity coincide with the minimal values of the entropy. As the strongly entangled states are produced, the linear entropy decreases. This relation is better seen in the map exhibiting the relations between negativity and linear entropy for all spin pairs—see Figure 11b. The gray dots represent results for spin pairs 1–2 and 4–5. For these two pairs, strong entanglement can occur. The red dots correspond to the results for other spin pairs for which the produced entanglement is weaker. From the figure, we see that the state’s mixedness decreases with increasing negativity. It suggests that strongly mixed states are weakly entangled or separated. In the case discussed here, the negativity does not exceed 0.2 for the states whose mixedness reaches its maximum value (0.7). This observation provides a quantitative characterization of entanglement limitations.

**Figure 12:**
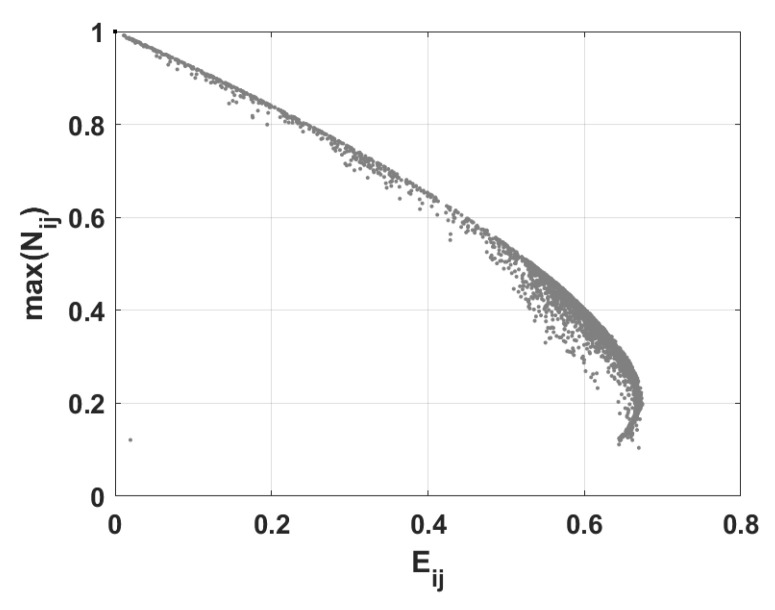
The map of $maxN_{ij}$ versus $E_{ij}$ for all spin pairs. The parameters are identical to those for Figure 3.

Analogously to the case illustrated in Figure 11b, Figure 12 presents a map exhibiting the maximal values of negativity and corresponding to linear entropy. Figure 12 corresponds to the scenario presented in Figure 3 in which the maximal values of the negativity were depicted. From Figure 12, we can see that the maximal value of negativity depends on the value of linear entropy. As the linear entropy increases, corresponding maximal values of negativity decrease in general. At this point, one should stress that although Figure 12 corresponds to the case depicted in Figure 3, similar behavior can be observed in all previously analyzed scenarios depicted in Figure 4, Figure 5, Figure 6, Figure 7, Figure 8, Figure 9 and Figure 10, which is consistent with the general constraints imposed by mixedness.

## 6. Conclusions

In this paper, we have studied the transfer and generation of entanglement in the spin-Ising system. The system consists of six spins that interact with one another to form a hexagon. Additionally, the spins are exposed to an external uniform magnetic field. We have considered two cases: (I) where two spins were initially prepared in the maximally entangled state, and (II) where the system was initially in the entirely separable state. In the first case, we analyzed how entanglement (quantified by the negativity) is transferred from the initially maximally entangled pair of spins to other pairs. In the second case, we studied the possibility of entanglement generation. To analyze the transfer and creation of entanglement, we used negativity.

In both scenarios, it has been demonstrated that the coupling parameters influence the transfer and generation of entanglement. By a proper manipulation of the values of the couplings, it is possible to create or transfer strong entanglement between any pair of spins. On the other hand, if the coupling strength is chosen incorrectly, the strong entanglement may not be produced or transferred.

Our results demonstrate that a deliberate tuning of the nearest-neighbor coupling strengths provides an effective and versatile tool for steering pairwise entanglement across the ring. In particular, we show that strong entanglement (with negativity exceeding 0.9) can be selectively established between specific spins, while simultaneously suppressing correlations in other pairs. This controllable redistribution mechanism distinguishes our approach from earlier studies of homogeneous chains, noncollinear interactions, and environmentally induced dynamics, as reported in [45,46,49]. Previous studies have emphasized different geometries, interaction types, and open-system effects. Here, in contrast, we provide a systematic analysis of entanglement engineering in a strictly finite, isolated, Ising-like ring.

Finally, we have shown that the production of strongly entangled two-spin states is associated with the disappearance of mixedness of such states (quantified by the linear entropy).

## Data Availability

The raw data supporting the conclusions of this article will be made available by the authors on request.
